# Differential effects of aging, Alzheimer's pathology, and *APOE4* on longitudinal functional connectivity and episodic memory in older adults

**DOI:** 10.1186/s13195-025-01742-6

**Published:** 2025-04-25

**Authors:** Larissa Fischer, Jenna N. Adams, Eóin N. Molloy, Niklas Vockert, Jennifer Tremblay-Mercier, Jordana Remz, Alexa Pichet Binette, Sylvia Villeneuve, Anne Maass, PREVENT-AD Research Group

**Affiliations:** 1 German Center for Neurodegenerative Diseases (DZNE), Magdeburg, Germany; 2 Department of Neurobiology and Behavior, University of California, Irvine, USA; 3 Department of Radiology & Nuclear Medicine, Faculty of Medicine, Otto Von Guericke University, Magdeburg, Germany; 4 StoP-AD Centre, Douglas Mental Health Institute Research Centre, McGill University, Montréal, Canada; 5 Department of Physiology and Pharmacology, Université de Montréal, Montréal, Canada; 6 Centre de Recherche de l’Institut Universitaire de Gériatrie de Montréal, Montréal, Canada; 7 Department of Clinical Sciences Malmö, Clinical Memory Research Unit, Lund University, Lund, Sweden; 8 Department of Psychiatry, McGill University, Montréal, Canada; 9 Institute for Biology, Otto Von Guericke University, Magdeburg, Germany

**Keywords:** Aging, Alzheimer’s disease, FMRI, Functional connectivity, Episodic memory, *APOE*

## Abstract

**Background:**

Both aging and Alzheimer's disease (AD) affect brain networks, with early disruptions occurring in regions involved in episodic memory. Few studies have, however, focused on distinguishing region-specific effects of AD-biomarker negative “normal” aging and early amyloid- and tau pathology on functional connectivity. Further, longitudinal studies combining imaging, biomarkers, and cognition are rare.

**Methods:**

We assessed resting-state functional connectivity (rsFC) strength and graph measures in the episodic memory network including the medial temporal lobe (MTL), posteromedial cortex (PMC), and medial prefrontal cortex alongside cognition over two years. For this preregistered study, we included 100 older adults who were amyloid- and tau-negative using CSF and PET measurements to investigate “normal” aging, and 70 older adults who had longitudinal CSF data available to investigate functional changes related to early AD pathology. All participants were cognitively unimpaired older adults from the PREVENT-AD cohort. We used region of interest (ROI)-to-ROI bivariate correlations, graph analysis, and multiple regression models.

**Results:**

In the amyloid- and tau-negative sample, rsFC strength within PMC, between parahippocampal cortex and inferomedial precuneus, and between posterior hippocampus and inferomedial precuneus decreased over time. Additionally, we observed a longitudinal decrease in global efficiency. Further, there was a steeper longitudinal decrease in rsFC and global efficiency with higher baseline age particularly of parahippocampal-gyrus regions. Further, lower rsFC strength within PMC was associated with poorer longitudinal episodic memory performance. In the sample with available CSF data, a steeper increase in rsFC between anterior hippocampus and superior precuneus was related to higher baseline AD pathology. Higher MTL-PMC rsFC strength was differentially associated with episodic memory trajectories depending on *APOE4* genotype.

**Conclusions:**

Our findings suggest differential effects of aging and AD pathology. Hypoconnectivity within PMC was related to aging and cognitive decline. MTL-PMC hyperconnectivity was related to early AD pathology and cognitive decline in *APOE4* carriers. Future studies should investigate more diverse samples, nonetheless, our approach allowed us to identify longitudinal functional changes related to aging and early AD pathology, enhancing cross-sectional research. Hyperconnectivity has been proposed as a mechanism related to early AD pathology before, we now contribute specific functional connections to focus on in future research.

**Graphical Abstract:**

**A**) “Normal aging” in cognitively unimpaired older adults with a negative amyloid- and tau- biomarker status was characterized by a longitudinal decrease in functional connectivity strength. **B**) Cognitively unimpaired older adults with more Alzheimer’s pathology at baseline (measured via cerebrospinal fluid) exhibited a longitudinal increase in functional connectivity strength.

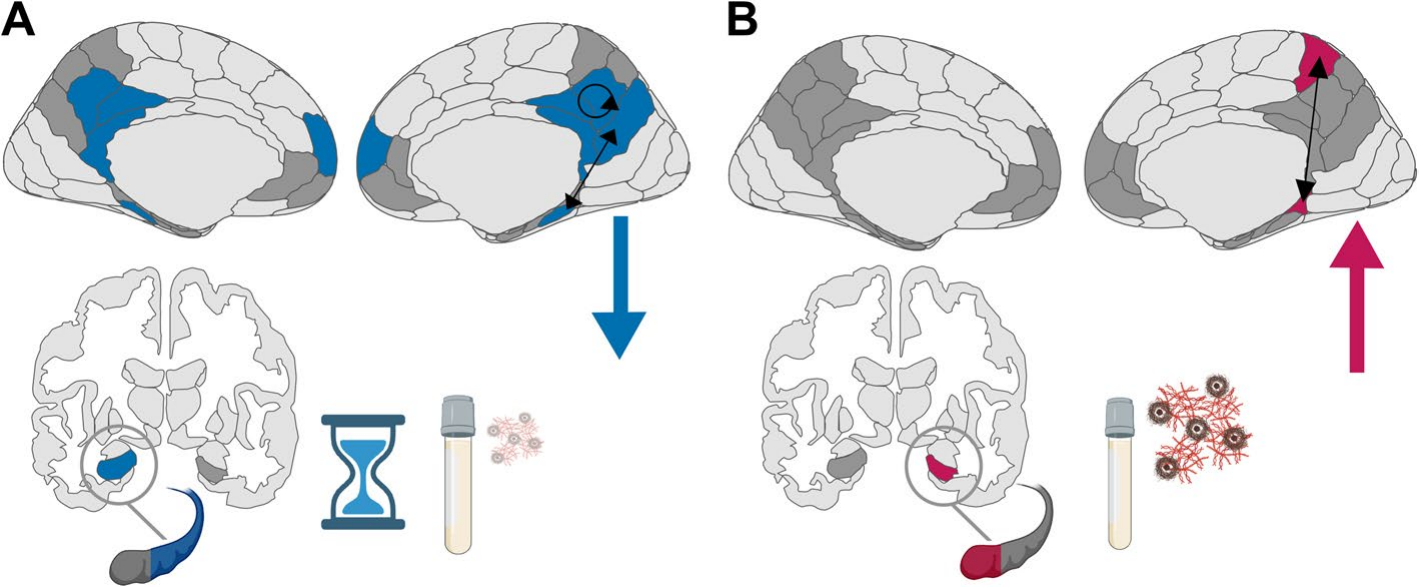

**Supplementary Information:**

The online version contains supplementary material available at 10.1186/s13195-025-01742-6.

## Introduction

Episodic memory deficits in aging and Alzheimer’s disease (AD) are hypothesized to partly result from network disruptions involving the medial temporal lobe (MTL) and neocortical brain regions [1–3]. Although intact episodic memory is key for independent living, it is among the first cognitive functions to deteriorate with age and AD [4, 5]. Functional connectivity (FC) of the episodic memory network, which includes the MTL, the posteromedial cortex (PMC) and the medial prefrontal cortex (mPFC), is crucial for remembering prior experiences [6–8], with the MTL being important for encoding [9], and the PMC being important for retrieval [10]. Especially FC within and between the MTL and PMC seems to be successible to early changes with aging and AD [11]. These changes in FC are, however, complex and poorly understood. Differentiating the respective functional dynamics offers significant potential for early identification of individuals in (future) need of clinical intervention and for targeting aberrant FC processes as intervention pathways [12, 13].

AD pathology includes amyloid-beta (Aβ) plaques and tau tangles, starting in the PMC [14] and the MTL [15], respectively, and is present in a substantial proportion of cognitively unimpaired older adults [16–18]. Given the importance of those brain regions for episodic memory function, understanding specific pathology-related network dynamics is of high interest. Aberrant functional network properties have been associated with Aβ [19–21] and tau [22–25] in cognitively unimpaired older adults. More specifically, animal models [26–28] and human studies [21, 23, 29–31] suggest that amyloid is rather related to hyperconnectivity, while tau may be first related to hyper- and then hypoconnectivity in later AD stages. Hyper- and hypoconnectivity are terms that describe deviant FC and are conceptually related to the phenomenon of hyper- and hyperactivation. These were predominantly described cross-sectionally relative to a control group using resting-state (rs) and task-based functional magnetic resonance imaging (fMRI) [32, 33]. Furthermore, graph-based studies have reported that cognitively impaired AD patients show disruptions and reduced centrality of hub regions in the default mode network (DMN), which largely overlaps with episodic memory network regions, compared to cognitively unimpaired individuals [34, 35].

While animal models can provide direct evidence for hyperexcitability from cell recordings [36], aberrant FC in fMRI studies is observed indirectly via changes in the co-variation in blood oxygen level-dependent (BOLD) signal between regions and changes in derived network properties. Pinning down brain areas within the episodic memory network that show the earliest FC changes in association with AD pathology remains a challenge. Regarding the MTL, cross-sectional studies suggested that the parahippocampal gyrus and the hippocampus are among the first MTL regions that show functional alterations with early AD pathology and with decline in memory function [37, 38]. Given their role as hub regions within the episodic memory network, these changes align with the hypothesis of hub hyperconnectivity in response to adverse influences [39]. Specific changes in network properties, like increased local efficiency of the DMN and the hippocampus, have further been linked to better memory in amyloid-positive cognitively normal individuals, suggesting beneficial or compensatory mechanisms [19]. Interestingly, especially the anterior hippocampus, entorhinal and perirhinal cortex seem to be involved in early amyloid-related hyperconnectivity [40–42].

Hypoconnectivity, on the other hand, has been cross-sectionally described in amyloid-negative relative to amyloid-positive individuals [40] and reduced DMN network integrity has been reported in aging studies [43]. Little is known, however, about how FC changes in cognitively unimpaired older adults with a negative amyloid- and tau- biomarker status. Most longitudinal studies investigating cognitively unimpaired older adults did not assess AD pathology or AD-risk factors like the Apolipoprotein e4 (*APOE4*) genotype, leaving significant uncertainty about the effects of aging in the absence of Aβ and tau [32]. As a result, it remains unclear which specific age-related and AD-related changes in FC can be distinguished, and how these changes relate to episodic memory performance.

To disentangle episodic memory network changes related to aging independent of AD pathology and neurodegenerative pathogenesis, we quantified longitudinal changes in rsFC in a group of cognitively unimpaired older adults (a) with a negative amyloid- and tau biomarker status and (b) with available longitudinal AD biomarker data (independent of biomarker status). Specifically, we aimed to relate rsFC changes to aging, Aβ and tau pathology, *APOE4* genotype, and longitudinal memory performance in the PREVENT-AD cohort [44] of cognitively unimpaired older adults with this preregistered study [45].

We hypothesized for the episodic memory network that

i) decreasing rsFC strength over time, decreasing integration of hub regions, and decreasing meaningful subnetwork segregation, especially in older age, is visible in individuals with low pathology (A^−^T^−^).
ii) increasing rsFC, decreasing integration of hub regions, and decreasing meaningful subnetwork segregation is associated with higher early AD pathology, especially in *APOE4* carriers.
iii) mild age-related (A^−^T^−^) decline or less practice effects in episodic memory performance are associated with steeper decrease in rsFC strength over time, especially in older age.
iv) higher or increasing rsFC strength could be an initial beneficial or compensatory process if predicting maintained episodic memory performance or a detrimental process if predicting decline in performance.

## Methods

### Sample and study design

All participants were cognitively unimpaired older adults from the Pre-symptomatic Evaluation of Experimental or Novel Treatments for Alzheimer’s Disease (PREVENT-AD) cohort [44, 46]. PREVENT-AD is an ongoing longitudinal study with first enrollments starting in 2011. The data we used were part of data release 7.0 with parts of the data being publicly available. Participants self-reported having a parental history of AD dementia or two siblings diagnosed with AD dementia and were above 60 years of age at enrollment. Participants aged between 55 and 59 were also included if their age was less than 15 years away from their affected relative’s age at onset. Participants had no major neurological or psychiatric illnesses at baseline and were screened for normal cognition with the Montreal Cognitive Assessment (MoCA) questionnaire [47] and the Clinical Dementia Rating (CDR) scale [48]. If a participant’s cognitive status was in doubt (MoCA < 27 or CDR > 0), the participant had to undergo exhaustive neuropsychological evaluation and was excluded upon a diagnosis of probable MCI.

We formed two samples to answer our specific research questions of dissociable effects of AD-independent aging and AD pathology. Sample A consisted of Aβ- and tau-negative older adults (A^−^T^−^, *N* = 100, 63 ± 6 years, 72 female, 30 *APOE4* carriers) with MRI scanning at baseline and follow-up sessions after 12 (FU12) and 24 months (FU24). We used PET and CSF data to evaluate biomarker status for Aβ (A^±^) and tau (T^±^). The use of both modalities for exclusion of biomarker-positive individuals allowed us to enlarge our sample, as 14 participants had CSF but no PET measurements and 78 had PET but no CSF measurements. Participants had to be biomarker-negative via CSF at FU24 or via PET, which took place on average 6 years after baseline. Any CSF data (if available) beyond FU24 also had to be negative. Sample B consisted of all older adults with available longitudinal CSF measurements (*N* = 70, 63 ± 5 years, 48 female, 24 *APOE4*), independent of biomarker status. The participants included in sample B had MRI and CSF measurements at baseline and at FU24. As four participants in sample B did not have complete data for FU12 available, we did not investigate this session. Thirty-nine participants were included (i.e. overlapping) in both samples.

After preprocessing of the MRI data, 8 participants with excessive motion were excluded during MRI quality control. Specifically, seven participants in sample A and one participant in sample B were excluded from further analysis due to more than 20% of volumes flagged as motion outliers (see Sect."MRI data acquisition and preprocessing"for details). This resulted in a final sample A of 93 older adults (A^−^T^−^, 63 ± 5 years, 68 female, 28 *APOE4* carriers) and sample B of 69 older adults (62 ± 6 years, 48 female, 23 *APOE4* carriers), see Table 1.

**Table 1:** Final sample demographics after exclusions

Sample	N	Age in years	Education in years	Female sex	*APOE4* carrier	CSF Aβ_1–42_ at baseline	CSF Aβ_1–42_ at FU24	CSF p-tau_181_ at baseline	CSF p-tau_181_ at FU24	Aβ PET SUVR	Tau PET SUVR
A	93	63.26 (4.69)	15.91 (3.58)	68 (73%)	28 (30%)	*N* = 46 1191.96 (183.86)	*N* = 46 1206.52 (202.01)	*N* = 40 40.52 (14.64)	*N* = 40 40.13 (14.46)	*N* = 78 1.174 (0.09)	*N* = 78 1.026 (0.09)
B	69	62.71 (5.64)	15.13 (2.97)	48(70%)	23 (33%)	*N* = 69 1142.06 (264.85)	*N* = 69 1150.10 (284.29)	*N* = 69 47.25 (19.37)	*N* = 69 47.50 (19.77)	*N* = 45 1.260 (0.22)	*N* = 45 1.032 (0.08)

Mean (standard deviation) or number (percentage) of participants. Thirty-nine participants were included in both samples. Age denotes age at baseline. All participants identified as either female or male. Two *APOE4* carriers in sample A were homozygous; there were no homozygous *APOE4* carriers in sample B. CSF measures are given in pg/mL and PET score in mean SUVR. For tau PET, entorhinal tau was investigated. For Aβ PET, neocortical global Aβ was used. *N* Number, *APOE4* Apolipoprotein e4, *CSF* Cerebrospinal fluid, *Aβ* Amyloid-beta, *PET* Positron emission tomography, *SUVR* Standardized uptake value ratio

**Fig. 1:**
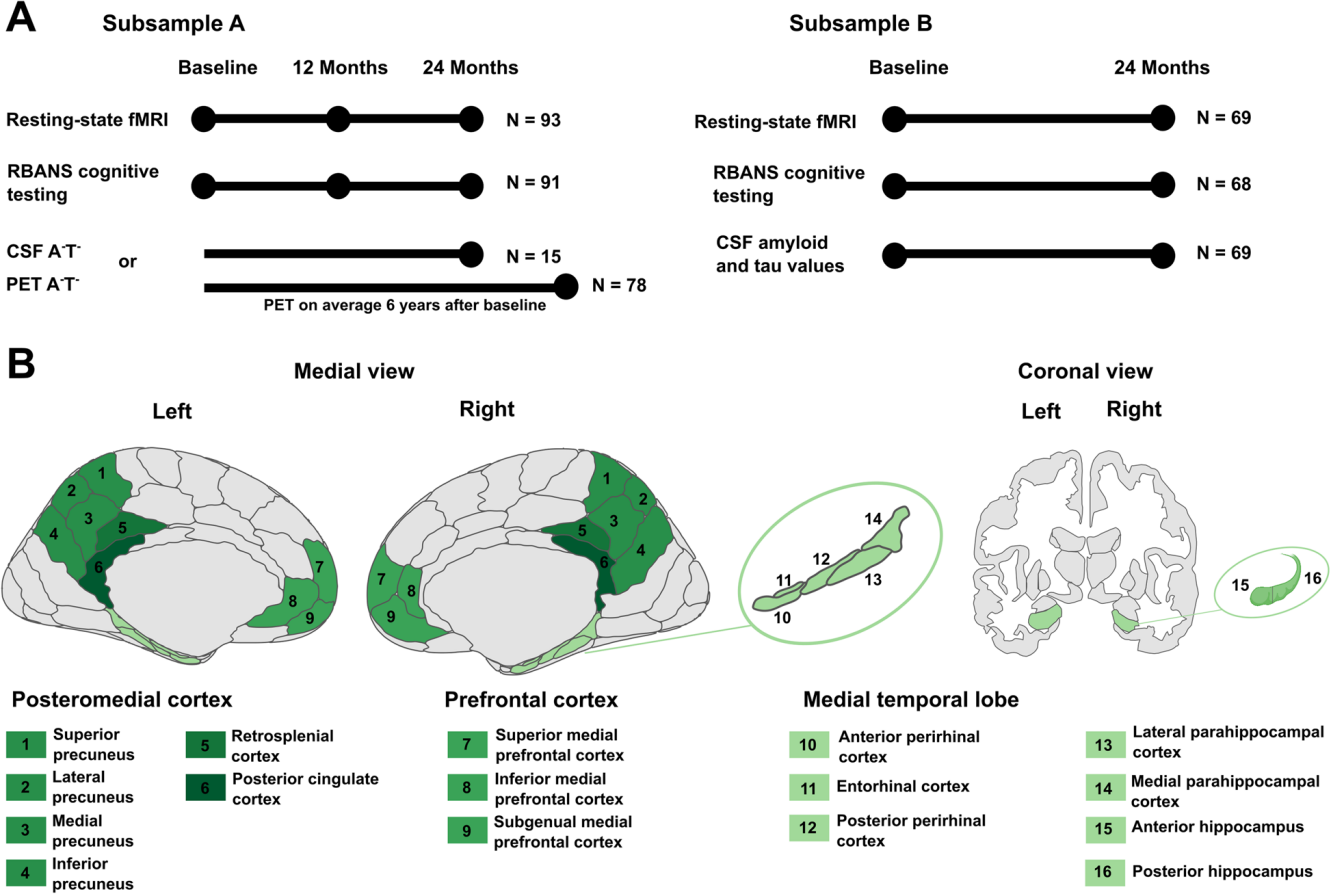
Study design and regions of interest.** A** Visualization of included sessions. Sample A consisted of 93 amyloid- and tau-negative (A^−^T^−^) older adults. Biomarker status was determined via CSF or PET. Participants had to be biomarker-negative via CSF at the last session of MRI and cognitive assessment we investigated, which was FU24. If only PET was available, they had to be negative at this measurement, which took place on average 6 years after baseline. If PET and CSF for sessions later than 24 months were available, both had to be below the respective threshold. Sample B consisted of 69 older adults with available longitudinal CSF measurements independent of biomarker status. **B** Visualization of regions of interest. Illustrations are presented in neurological view. Brain regions considered as subregions of a larger region (precuneus, medial prefrontal cortex, and medial temporal lobe) are presented in the same shade of green. Retrosplenial cortex and posterior cingulate cortex were not further divided into subregions. Medial view is based on the Brainnetome atlas, which was used for ROI definition. The coronal view is based on the aseg atlas for illustration of the hippocampus only. fMRI = functional magnetic resonance imaging. CSF = cerebrospinal fluid. PET = positron emission tomography. RBANS = Repeatable Battery for Assessment of Neuropsychological Status

All study procedures and experimental protocols were approved by the McGill University Institutional Review Board and/or the Douglas Mental Health Institute Research Ethics Board in accordance with the Declaration of Helsinki. Written informed consent was obtained from all participants prior to each experimental procedure and they were financially compensated for their time. For an overview of the included sessions, see Fig. 1A.

### Cerebrospinal fluid collection

Participants who consented to this procedure contributed CSF samples from a lumbar puncture (LP), as described by Tremblay-Mercier and colleagues [44]. The LP took place on average 28 days after cognitive assessment and fMRI. A large-bore introducer was inserted at the L3-L4 or L4-L5 intervertebral space, then an atraumatic Sprotte 24 ga. spinal needle was used to puncture the dura. Up to 30 ml of CSF were withdrawn in 5.0 ml polypropylene syringes, centrifuged at room temperature for 10 min at ~ 2000 g, aliquoted in 0.5 ml polypropylene cryotubes, and quick-frozen at − 80 °C for long-term storage. CSF measurements of interest for this study were Aβ_1–42_ and p-tau_181_ and the p-tau_181_/Aβ_1–42_ ratio to assess the effect of AD pathology. The ratio is an established measure in AD research [49, 50] and was included to assess the combined effect of amyloid-related tau hyperphosphorylation. Biomarker status was evaluated via research cut-offs defined by the PREVENT-AD Research Group, namely CSF Aβ_1–42_ < 850 pg/ml, CSF p-tau_181_ > 60 pg/ml.

### *APOE* genotyping

All included participants were genotyped for *APOE* via a QIASymphony apparatus. For details, see the description by Tremblay-Mercier and colleagues [44]. Participants who featured at least one copy of the *APOE4* allele were included in the carrier group while participants without an *APOE4* allele were allocated to the non-carrier group.

### Assessment of episodic memory performance

Cognition was measured via the longitudinal Repeatable Battery for Assessment of Neuropsychological Status (RBANS) at each session [51]. Different versions of the RBANS were used at each follow-up session to reduce practice effects. Our main measure of interest was the RBANS delayed memory index score, which integrates assessments from word-list recognition as well as delayed free recall of figures, stories, and word lists. To investigate the specificity of the associations between longitudinal memory performance and rsFC, we also used the attention index score as a memory-independent measure in an exploratory control analysis. The attention index score consists of two digit span subtests. RBANS data from baseline and FU24 were used to investigate the change in performance over two years. Baseline RBANS data were not available for three participants in sample A and two participants in sample B, for whom we used the RBANS data measured three months after baseline. Follow-up RBANS data were available for all participants. Two participants in sample A and one participant in sample B were excluded from the investigation of cognition, as there were indications of possible mild cognitive impairment (MCI) between one and three years after baseline.

### MRI data acquisition and preprocessing

MRI data were acquired on a Siemens Tim Trio 3-Tesla MRI scanner at the Cerebral Imaging Centre of the Douglas Mental Health University Institute using a standard 12- or 32-channel coil (Siemens Medical Solutions, Erlangen, Germany). T1-weighted anatomical MPRAGE scans (TR = 2300 ms; TE = 2.98 ms; TI = 900 ms; 9° flip angle; FOV = 256 × 240x176 mm) were acquired with a 1 mm isotropic voxel resolution. Resting-state data over 10 min were obtained using an EPI sequence (TR = 2000 ms; TE = 30 ms; a = 90◦; FOV = 256 × 256 mm; 32 slices) with a 4 mm isotropic voxel resolution. Fieldmaps were acquired to correct for spatial distortions of the functional data due to field inhomogeneities during unwarping.

Functional and structural data were preprocessed using MATLAB, Statistical Parametric Mapping, version 12 (SPM12) [52] and the CONN toolbox, version 22.a [53]. Voxel-displacement maps were created using fieldmaps from the respective scanning session to correct susceptibility artifacts via unwarping. The fMRI data were slice-time corrected, realigned to the first volume of the first session and co-registered to the structural T1-weighted images of the respective session. The structural images were segmented into gray matter, white matter, and cerebrospinal fluid and normalized to the Montreal Neurological Institute (MNI) reference frame using the IXI549Space template. The translation parameters derived from the structural images were then applied to the fMRI data for normalization to MNI space. Outlier fMRI volumes due to excessive head motion were detected using ART [54], where volumes with framewise displacement above 0.5 mm/TR or a global intensity z-score of 3 were flagged. fMRI sessions with more than 20% of volumes flagged as outliers were excluded from the analysis (see Sect."Sample and study design"for details). During denoising of fMRI data, confounding effects were regressed out using realignment parameters and their first order derivatives, flagged outlier volumes, and signal from cerebral white matter and cerebrospinal fluid derived via anatomical CompCor [55, 56]. A band-pass filter of 0.008 Hz to 0.09 Hz was applied to minimize noise from physiological and motion sources [57].

We additionally utilized FreeSurfer 7.1 (Laboratory for Computational Neuroimaging, Athinoula A. Martinos Center) and the longitudinal pipeline [58, 59] to derive hippocampus volume over time. T1-weighted MRI data for each session were processed using the standard cross-sectional pipeline to generate anatomical reconstructions. An unbiased within-subject template was then created from the images of all three sessions (baseline, FU12, FU24). One participant had no MRI data for FU12 available. Hippocampal volumes were then extracted from the segmented images for each session, registered to the within-subject template.

### Functional connectivity analysis

Regions of interest (ROIs) from the Brainnetome atlas [60] were defined a priori based on a literature research on episodic memory brain areas. They include i) the MTL regions anterior and posterior hippocampus, lateral and medial parahippocampal cortex, entorhinal cortex and anterior and posterior perirhinal cortex; ii) the PMC regions precuneus, posterior cingulate cortex and retrosplenial cortex and iii) mPFC. The precuneus consisted of four subregions, the mPFC of three subregions. For a visualization of ROIs, see Fig. 1B. As a control network, we used the visual network from the Yeo atlas [61]. For all atlas labels see Supplementary Table S1.

During first-level analysis, connectivity matrices were derived for each individual participant and session. Fisher z-transformed correlation coefficients for each ROI pair were computed by bivariate correlations. Convolution of the equal weights of the scans with a canonical hemodynamic response function (hrf-weighting) was applied.

During second-level analysis, a separate General Linear Model (GLM) was estimated for each individual functional connection. Inferential statistics were performed at the network level (i.e. clusters of connections) and based on the hypothesis defining the expected association. Network-level inferences were based on nonparametric statistics from Network Based Statistics analyses (NBS) which uses randomization (10,000 iterations) of residuals from the second-level model to obtain uncorrected network-level *p*-values. Results were thresholded using a combination of a cluster-forming connection-level threshold of *p* < 0.001 and a Benjamini-Hochberg [62] false-discovery rate (FDR)-corrected network-level threshold of *p*-FDR < 0.05 [53].

### Graph analysis

Graph analysis was conducted to assess changes in network properties over time [63, 64]. A graph consists of nodes and edges (connections) and represents the elements of a complex system and their interrelationships [65]. We focused on two main aspects: episodic memory network integration and subnetwork segregation (i.e. MTL, PMC and mPFC separately). For network integration, we calculated global efficiency, shortest path length, and nodal betweenness centrality. Global efficiency was measured to assess the efficiency of information transfer between nodes. Shortest path length was analyzed to determine the average minimum distance between nodes, with an increase indicating reduced network efficiency. Nodal betweenness centrality was used to identify how central specific nodes are to the network's communication pathways. For subnetwork segregation, we focused on the clustering coefficient and local efficiency. The clustering coefficient was measured to evaluate the extent of local clustering within the network, indicating how well nodes form tightly-knit subgroups. Local efficiency was calculated to assess the efficiency of information transfer within these local subnetworks.

We specified in our preregistration [45] to graphically determine the optimal primary threshold for each sample [66](see Supplementary Figure S1 for details). In short, we calculated global and local efficiency across cost thresholds for each sample, comparing the measures for the real data to random and lattice networks. We used the formula *Global efficiency (Data)—Global efficiency (Lattice)* + *Local efficiency (Data)—Local efficiency (Random)*. The highest value, representing the greatest divergence between the real network and the modeled structures, was selected as the optimal cost threshold. This resulted in a cost threshold of 0.14 for sample A and 0.16 for sample B, which were then applied to the respective second-level rsFC matrix to establish the binarized adjacency matrix. A second-level Benjamini–Hochberg FDR-corrected threshold of *p*-FDR < 0.05 was applied.

### PET data acquisition and preprocessing

Positron emission tomography (PET) imaging was conducted at the McConnell Brain Imaging Centre of the Montreal Neurological Institute (Quebec, Canada) utilizing a brain-dedicated Siemens PET/CT high-resolution research tomograph. Data acquisition and processing followed the methodology described by Yakoub and colleagues [67]. Briefly, Aβ-PET images using 18 F-NAV4694 (NAV) were acquired between 40 and 70 min post-injection, with an approximate injection dose of 6 mCi. Tau-PET images, utilizing 18 F-flortaucipir (FTP), were captured 80 to 100 min post-injection, with an injection dose of around 10 mCi. The scans included 5-min frames and an attenuation scan.

PET images were reconstructed using a 3D ordinary Poisson ordered subset expectation maximum algorithm (OP-OSEM) with 10 iterations and 16 subsets, and were corrected for decay and motion. Scatter correction employed a 3D scatter estimation method. T1-weighted MRI images were segmented into ROIs based on the Desikan-Killiany atlas using FreeSurfer version 5.3. PET images were then realigned, temporally averaged, and co-registered to the participants’ corresponding T1-weighted image, specifically the scan closest in time to PET data acquisition. These images were then masked to exclude CSF signal and smoothed with a 6 mm Gaussian kernel. Standardized uptake value ratios (SUVRs) were calculated as the ratio of tracer uptake in the ROIs relative to the cerebellar gray matter for amyloid-PET scans, or to the inferior cerebellar gray matter for tau-PET scans. All PET data preprocessing was conducted using a standard pipeline available at https://github.com/villeneuvelab/vlpp. We assessed bilateral entorhinal FTP SUVR by averaging the uptake ratios of the left and right entorhinal cortices and global neocortical NAV SUVR consisting of bilateral lateral and medial frontal, parietal, and lateral temporal regions. Biomarker status was evaluated based on research cut-offs defined by the PREVENT-AD Research Group, namely entorhinal FTP SUVR > 1.3 and global neocortical NAV SUVR > 1.39.

### Statistical analysis

Statistical analysis was conducted with the CONN toolbox and R [68] version 4.2.3 using RStudio, version 2022.07.1 [69]. Figures were created using the packages ggplot [70] and ggseg [71]. The R code used for analyses is publicly available (https://github.com/fislarissa/MTL_PMC_longitudinal_rsFC). For linear models, we ensured that multicollinearity and heteroscedasticity were not present. Further, we tested for a normal distribution of residuals using the Shapiro–Wilk test on the standardized residuals. For individual connections that were part of a significant cluster, Cohen’s d (d) for t-tests and standardized β for regressions with the corresponding 95% confidence intervals (95% CI) were computed. For clusters, we calculated the 95% confidence interval for the (FDR-corrected) cluster *p*-values obtained from the permutation test (95% CI_*p*_) to quantify the uncertainty due to the finite number of permutations [72].

Measures of change were calculated as difference scores, i.e. *pathology at FU24—pathology at baseline and RBANS at FU24—RBANS at baseline*. Age, *APOE4* group, sex, education, and difference in days between baseline and the respective follow-up session were included as covariates.

The first hypothesis regarding the episodic memory network was that decreasing rsFC strength over time, decreasing integration of hub regions, and decreasing meaningful subnetwork segregation, especially in older age, is visible in individuals with low pathology (A^−^T^−^). Therefore, in sample A, we first investigated the i) FU12 > baseline and ii) FU24 > baseline contrast to identify connections or graph measures with significant change between sessions via paired t-tests in CONN. Second, we used change in rsFC and graph measures as dependent variables in multiple linear regression models with baseline age as independent variable. Third, we conducted an additional exploratory cross-sectional analysis of effects of baseline age on baseline rsFC.

The second hypothesis was that increasing rsFC, decreasing integration of hub regions, and decreasing meaningful subnetwork segregation is associated with higher early AD pathology, especially in *APOE4* carriers. Therefore, in sample B, we used change in rsFC and graph measures from baseline to FU24 as dependent variables in multiple linear regression models. As independent variables of interest, we used i) change of pathology from baseline to FU24 and ii) pathology at baseline. We formed separate models to investigate the effect of Aβ_1–42_, p-tau_181_, and the p-tau_181_/Aβ_1–42_ ratio.

Our two remaining hypotheses were centered around the association of longitudinal memory performance and rsFC strength (i) at baseline and (ii) over time. We first conducted paired t-tests in sample A and B to examine changes in memory performance, as measured by the RBANS delayed memory index score, over two years. We then extracted the first-level rsFC matrices from CONN for (i) the functional connection that showed the strongest decrease in rsFC strength over time (in cluster 1) over two years in sample A (i.e. retrosplenial cortex—posterior cingulate cortex) and (ii) the functional connection that showed the strongest relationship to AD pathology (within the significant cluster) over two years in sample B (i.e. anterior hippocampus—superior precuneus) to avoid multiple testing.

The third hypothesis was that mild age-related (A^−^T^−^) decline or less practice effects in episodic memory performance are associated with steeper decrease in rsFC strength over time, especially in older age. The fourth hypothesis was that higher or increasing rsFC strength could be an initial beneficial or compensatory process if predicting maintained episodic memory performance or a detrimental process if predicting decline in performance.

We predicted change in memory over two years with change in rsFC strength over two years in sample A and B separately. We then repeated these models while including the interaction terms rsFC*APOE and rsFC*age in sample A and rsFC*APOE and rsFC*p-tau_181_/Aβ_1–42_ ratio in sample B. These interaction terms were included exploratively based on prior findings [73]. To additionally address baseline rsFC strength as stated in the fourth hypothesis, we repeated these models with the respective measure of rsFC strength at baseline as predictor.

### Control analysis

First, as a planned control analysis, we used the Yeo visual network in the same models as described for the episodic memory network. Further, we exploratively included framewise-displacement motion estimates to all models in CONN.

Second, as an exploratory control analysis, we used change in the RBANS attention index score over two years as outcome variable in the same models as described for the RBANS delayed memory index score.

Third, as an exploratory control analysis, we investigated structural change in the whole bilateral hippocampus as a proxy for early neurodegenerative processes. For sample A and B separately, we first used a rmANOVA to test for changes in hippocampus volume over the three sessions and performed post hoc t-tests for a significant ANOVA. We then (i) correlated rate of change of hippocampus volume with AD pathology in sample B, (ii) predicted rate of change of hippocampus volume in linear models for sample A and B separately and (iii) repeated the linear models described above with inclusion of rate of change of hippocampus volume as an additional covariate. We used the normalized rate of change of hippocampus volume over two years ((volume at FU24—volume at BL)/volume at BL* 100).

## Results

### Assessment of change in rsFC strength and network properties over one and two years

In sample A (A^−^T^−^ individuals), rsFC decreased significantly over one year (from baseline to FU12) and over two years (from baseline to FU24), each time in two clusters of connections. Detailed statistics for all connections are provided in Table 2.

**Table 2:** Statistical reporting for longitudinal decrease in functional connectivity strength over time in amyloid- and tau-negative older adults

Cluster	Functional connection	t	df	Cohen’s d	95% CI
FU12 > baseline cluster 1					
	Left superior mPFC—right PCC	− 4.26	92	− 0.44	− 0.859, − 0.025
	Left superior mPFC—left PCC	− 3.62	92	− 0.38	− 0.791, 0.040
	Left superior mPFC—left RSC	− 3.47	92	− 0.36	− 0.775, 0.056
FU12 > baseline cluster 2					
	Left lateral PHC—left medial precuneus	− 4.33	92	− 0.45	− 0.866, − 0.032
	Left lateral PHC—right medial precuneus	− 3.36	92	− 0.35	− 0.764, 0.066
	Right lateral PHC—left medial precuneus	− 3.25	92	− 0.34	− 0.752, 0.078
FU24 > baseline cluster 1					
	Right RSC—right PCC	− 4.06	92	− 0.42	− 0.837, − 0.005
	Left RSC—right PCC	− 3.69	92	− 0.38	− 0.799, 0.033
	Left superior mPFC—right PCC	− 3.60	92	− 0.37	− 0.789, 0.042
	Right RSC—right lateral precuneus	− 3.58	92	− 0.37	− 0.787, 0.044
	Left superior mPFC—left PCC	− 3.43	92	− 0.36	− 0.770, 0.060
	Right RSC—right medial precuneus	− 3.22	92	− 0.33	− 0.748, 0.081
FU24 > baseline cluster 2					
	Left posterior hippocampus—right inferior precuneus	− 4.15	92	− 0.43	− 0.847, − 0.013
	Right lateral PHC—right inferior precuneus	− 4.01	92	− 0.42	− 0,833, 0.000
	Left posterior hippocampus—left medial precuneus	− 3.30	92	− 0.34	− 0.757, 0.073

For all functional connections that were part of significant clusters using the network-based statistics (NBS) approach, t-values (t), degrees of freedom (df), Cohen’s d, and the corresponding 95% confidence interval (CI) are reported. *FU12* Follow-up assessment after 12 months, *FU24* Follow-up assessment after 24 months, *mPFC* medial prefrontal cortex, *PCC* Posterior cingulate cortex, *RSC* Retrosplenial cortex, *PHC* Parahippocampal cortex

**Fig. 2:**
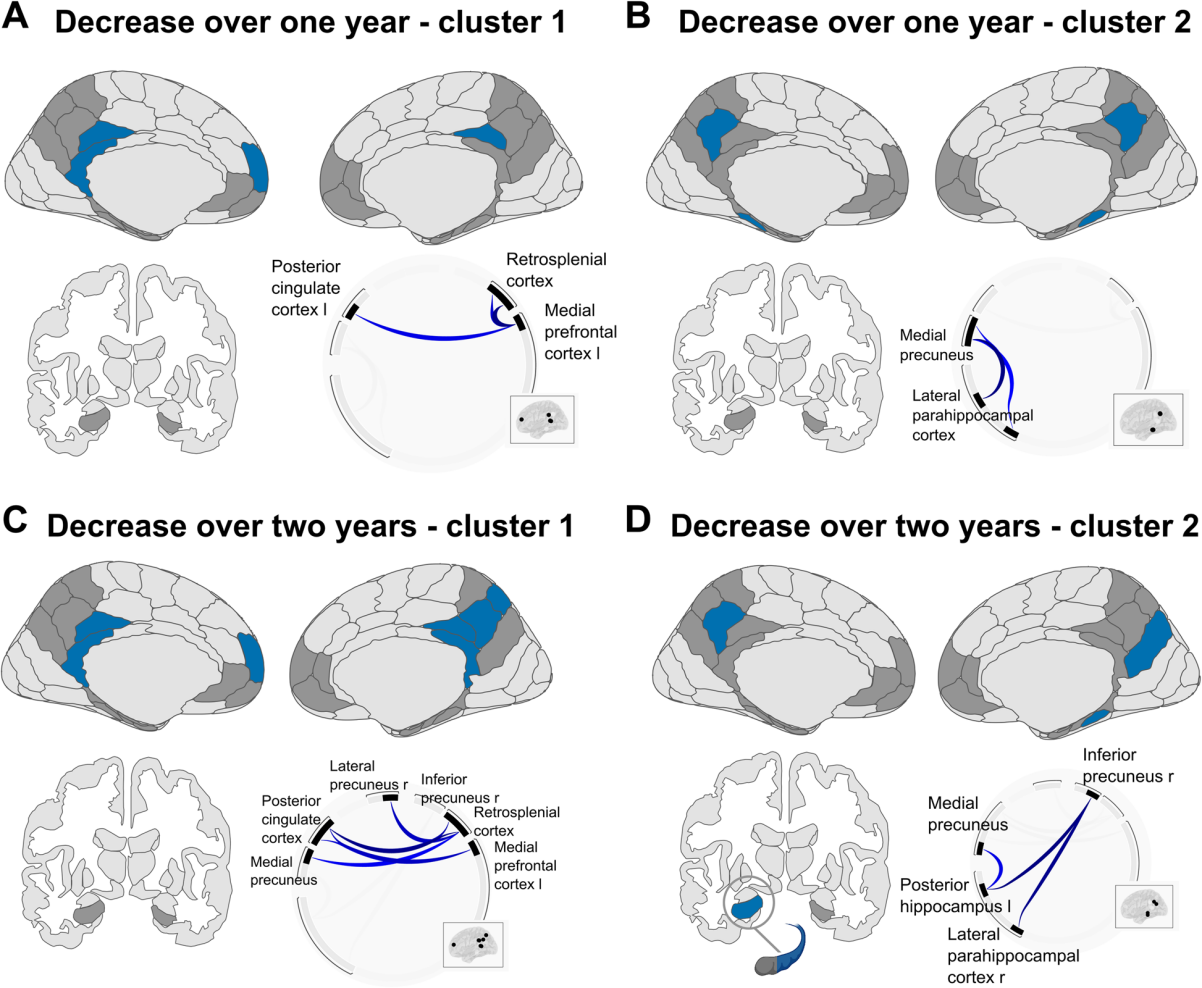
Longitudinal decrease in resting-state functional connectivity in amyloid- and tau-negative older adults. Illustrations are presented in neurological view. **A** Cluster 1 showing decrease in rsFC over one year (baseline to follow-up after 12 months). **B** Cluster 2 showing decrease in rsFC over one year (baseline to follow-up after 12 months). **C** Cluster 1 showing decrease in rsFC over two years (baseline to follow-up after 24 months). D) Cluster 2 showing decrease in rsFC over two years (baseline to follow-up after 24 months). l = left. r = right. See Table 2 for statistics

For baseline to FU12, cluster 1 (*p*-FDR = 0.008, [95%CI_*p*_ 0.006, 0.01], see Fig. 2A) consisted of the left superior mPFC and three regions of the PMC. Specifically, rsFC decreased between left superior mPFC—right posterior cingulate cortex, left superior mPFC—left posterior cingulate cortex, and left superior mPFC—left retrosplenial cortex. Cluster 2 (*p*-FDR = 0.008, [95%CI_*p*_ 0.006, 0.01], see Fig. 2B) consisted of the bilateral lateral parahippocampal cortex and the medial precuneus. Specifically, rsFC decreased between left lateral parahippocampal cortex—left medial precuneus, left lateral parahippocampal cortex—right medial precuneus, and right lateral parahippocampal cortex—left medial precuneus.

For baseline to FU24, cluster 1 (*p*-FDR = 0.001, [95%CI_*p*_ 0.001, 0.002], see Fig. 2C) consisted of the superior mPFC and ROIs within the PMC. Specifically, rsFC decrease between right retrosplenial cortex—right posterior cingulate cortex, left retrosplenial cortex—right posterior cingulate cortex, left superior mPFC—right posterior cingulate cortex, right retrosplenial cortex—right lateral precuneus, left superior mPFC—left posterior cingulate cortex, and right retrosplenial cortex—right medial precuneus. Cluster 2 (*p*-FDR = 0.005, [95%CI_*p*_ 0.003, 0.006], see Fig. 2D) consisted of the posterior MTL and the inferomedial precuneus. Specifically, rsFC decreased between left posterior hippocampus—right inferior precuneus, right lateral parahippocampal cortex—right inferior precuneus, and left posterior hippocampus—left medial precuneus.

In summary, we observed decreasing rsFC strength over one and two years in sample A (A^−^T^−^ individuals). This included decreasing rsFC between the mPFC and the PMC, between the MTL and the PMC and within the PMC subnetwork.

**Fig. 3:**
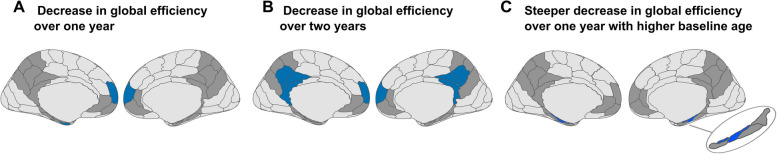
Longitudinal decrease in global efficiency in amyloid- and tau- negative older adults. Illustrations are presented in neurological view. As there was no involvement of the hippocampus, the coronal view is not shown. **A** Decrease in episodic-network global efficiency over one year, from baseline to the follow-up after 12 months, involving the left and right superior mPFC and left anterior PRC. **B** Decrease in episodic-network global efficiency over two years, from baseline to the follow-up after 24 months, involving the RSC, superior mPFC, PCC, and medial PCun, each in the left and right hemisphere. **C** Steeper longitudinal decrease in episodic-network global efficiency over one year with higher baseline age involving the posterior PRC and EC, each in the left and right hemisphere. mPFC = medial prefrontal cortex. PRC = perirhinal cortex. RSC = retrosplenial cortex. PCC = posterior cingulate cortex. PCun = precuneus. EC = entorhinal cortex

With respect to graph analyses, the only network property that showed significant longitudinal change over time in sample A (A^−^T^−^ individuals) was global efficiency, a graph measure of integration of nodes within a network. Global efficiency is the normalized average inverse shortest path-length of a node with each of the other nodes of the network. Global efficiency significantly decreased over one year (t(92) = − 1.86, *p* = 0.033) and marginally decreased over two years (t(92) = − 1.65, *p* = 0.051). Over one year, the nodes significantly contributing to the decrease in global efficiency were the bilateral superior mPFC and the left anterior perirhinal cortex (see Fig. 3A). Over two years, the nodes significantly contributing to the decrease were the retrosplenial cortex, superior mPFC, posterior cingulate cortex and medial precuneus, all bilateral (see Fig. 3B). Statistical reporting for each node can be found in Table 3.

**Table 3:** Statistical reporting for nodes contributing to longitudinal decrease in global efficiency over time in amyloid- and tau-negative older adults

Comparison of global efficiency	Node	t	df	*p*-FDR	Effect size	95% CI
FU12 > baseline						
	Left superior mPFC	− 4.02	92	0.002	d = − 0.42	− 0.833, − 0.000
	Right superior mPFC	− 2.72	92	0.048	d = − 0.28	− 0.696, 0.132
	Left anterior PRC	− 2.67	92	0.048	d = − 0.28	− 0.691, 0.137
FU24 > baseline						
	Right RSC	− 4.99	92	< 0.001	d = − 0.52	− 0.937, − 0.099
	Left superior mPFC	− 3.23	92	0.014	d = − 0.33	− 0.750, 0.080
	Left RSC	− 3.08	92	0.014	d = − 0.32	− 0.734, 0.095
	Right PCC	− 2.88	92	0.017	d = − 0.30	− 0.713, 0.115
	Right superior mPFC	− 2.83	92	0.017	d = − 0.29	− 0.707, 0.121
	Right medial precuneus	− 2.78	92	0.017	d = − 0.29	− 0.703, 0.125
	Left medial precuneus	− 2.69	92	0.019	d = − 0.28	− 0.693, 0.135
	Left PCC	− 2.59	92	0.022	d = − 0.27	− 0.682, 0.145

For all significant nodes, t-values (t), degrees of freedom (df), the false-discovery-rate corrected *p*-value (*p*-FDR), Cohen’s d (d) or standardized beta-values (beta), and the corresponding 95% confidence interval (CI) are reported. *FU12* Follow-up assessment after 12 months, *FU24* Follow-up assessment after 24 months, *mPFC* medial prefrontal cortex, *PRC* Perirhinal cortex, *RSC* Retrosplenial cortex, *PCC* Posterior cingulate cortex

### Assessment of the effects of baseline age on change in rsFC strength and network properties over one and two years

**Fig. 4:**
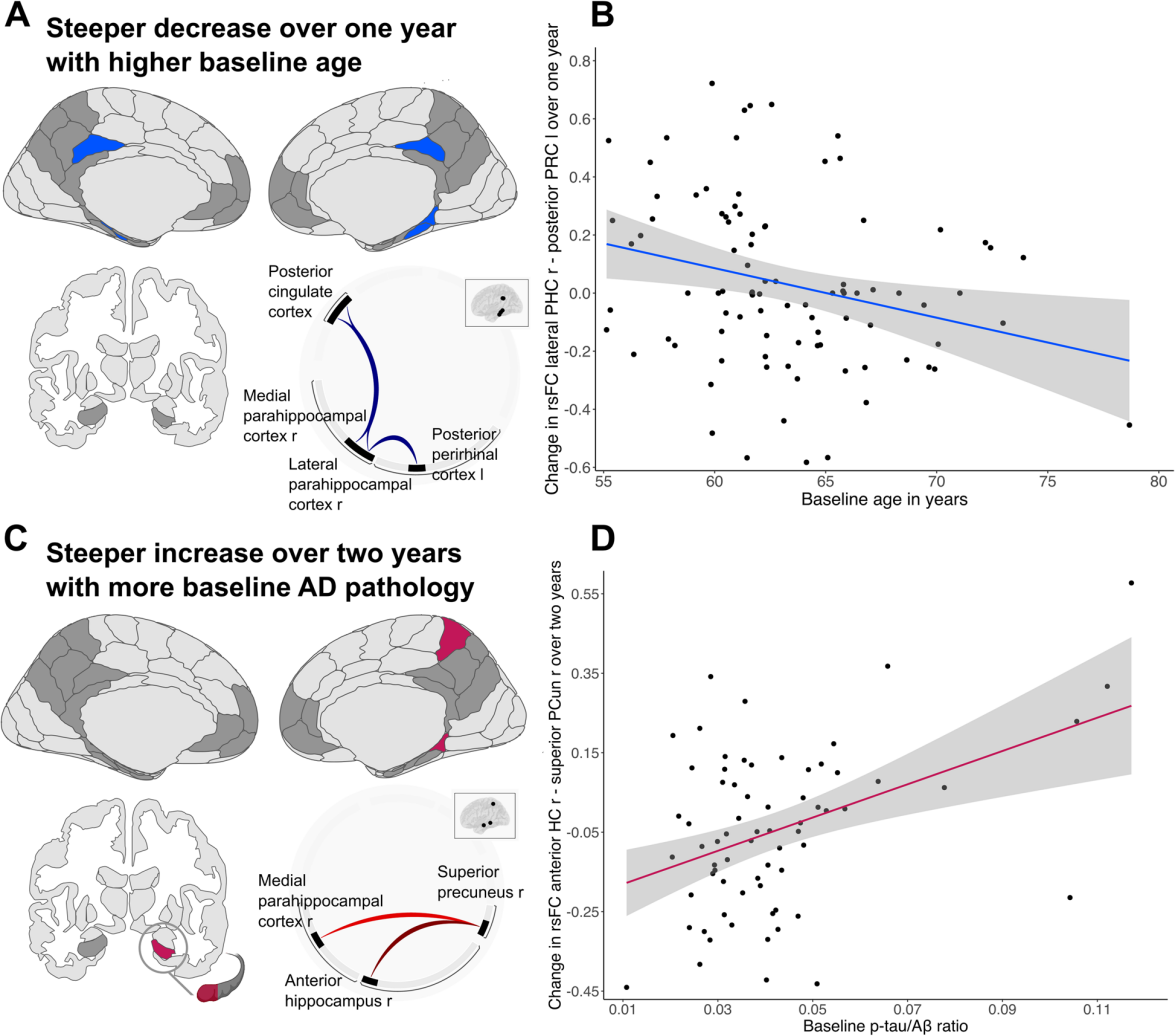
Longitudinal change in functional connectivity strength with higher baseline age and more baseline Alzheimer’s disease pathology. Illustrations are presented in neurological view. **A** In sample A (amyloid- and tau-negative older adults), there was a steeper longitudinal decrease in resting-state functional connectivity (rsFC) over one year with higher baseline age in the brain regions highlighted in blue. **B** Change in rsFC over one year was negatively associated with baseline age. The functional connection exhibiting the strongest effect (lateral parahippocampal cortex—posterior perirhinal cortex) is used for visualization. **C** In sample B (older adults with available longitudinal cerebrospinal fluid (CSF) measurements), there was a steeper longitudinal increase in rsFC over two years with a higher baseline p-tau_181_/Aβ_1–42_ ratio measured in CSF in the brain regions highlighted in red. **D** Change in rsFC over two years was positively associated with the p-tau_181_/Aβ_1–42_ ratio at baseline. The functional connection exhibiting the strongest effect (anterior hippocampus—superior precuneus) is used for visualization. l = left. r = right. rsFC = resting-state functional connectivity. PHC = parahippocampal cortex. PRC = perirhinal cortex. AD = Alzheimer’s disease. Aβ = amyloid-beta. HC = hippocampus. PCun = precuneus

Further, there was a significant effect of baseline age on change in rsFC in sample A over one year for a cluster including five ROIs (*p* = 0.034, [95%CI_*p*_ 0.030, 0.038], see Fig. 4A and Supplementary Tables S2 - 4), with involvement of the parahippocampal cortex in every connection. In this cluster, higher baseline age was related to a steeper decrease in rsFC over time. The specific connections were right lateral parahippocampal cortex—left posterior perirhinal cortex (β = − 0.34, t(87) = − 3.15, [95%CI − 0.553, − 0.125], see Fig. 4B), right medial parahippocampal cortex—right posterior cingulate cortex (β = − 0.32, t(87) = − 3.03, [95%CI − 0.537, − 0.112]), and right lateral parahippocampal cortex—left posterior cingulate cortex (β = − 0.33, t(87) = − 3.03, [95%CI − 0.541, − 0.112]).

There was no significant effect of baseline age on change in rsFC over two years (*p* > 0.05). There were no significant effects of *APOE4* group, sex, or education neither for change in rsFC from baseline to FU12 nor for baseline to FU24 (all *p* > 0.05). In an additional exploratory analysis, we did not find significant differences in baseline rsFC related to baseline age (*p* > 0.05).

With respect to graph analyses, there was an effect of baseline age on change in episodic-network global efficiency in sample A (A^−^T^−^ individuals) over one year (t(87) = − 2.21, *p* = 0.02) with the bilateral posterior perirhinal cortex and the bilateral entorhinal cortex (see Fig. 3C) significantly contributing, such that a stronger decrease in global efficiency was present in older A^−^T^−^ individuals. Statistical reporting for each node can be found in Table 4.

**Table 4:** Statistical reporting for nodes contributing to steeper longitudinal decrease in global efficiency with higher baseline age

Comparison of global efficiency	Node	t	df	*p*-FDR	Effect size	95% CI
Effect of baseline age on FU12 > baseline						
	Right posterior PRC	− 4.17	87	0.001	beta = − 0.43	− 0.642, − 0.227
	Left EC	− 3.71	87	0.003	beta = − 0.39	− 0.605, − 0.183
	Left posterior PRC	− 3.65	87	0.003	beta = − 0.38	− 0.594, − 0.175
	Right EC	− 2.71	87	0.047	beta = − 0.39	− 0.605, − 0.183

For all significant nodes, t-values (t), degrees of freedom (df), the false-discovery-rate corrected *p*-value (*p*-FDR), Cohen’s d (d) or standardized beta-values (beta), and the corresponding 95% confidence interval (CI) are reported. *FU12* Follow-up assessment after 12 months, *PRC* Perirhinal cortex, *EC* Entorhinal cortex

There was no significant effect of baseline age on change in global efficiency from baseline to FU24 and no effect of *APOE4* group on change in graph measures (all *p* > 0.05).

### Assessment of the effects of AD pathology on change in rsFC strength and network properties over two years

In sample B, there was no association of change in rsFC strength with change in Aβ_1–42_, p-tau_181_, or the p-tau_181_/Aβ_1–42_ ratio (all *p* > 0.05). For all three AD biomarker variables, we investigated change over two years, from baseline to FU24. None of the three biomarkers of AD pathology did change significantly over this timespan (all *p* > 0.05). We therefore investigated whether each biomarker at baseline showed an effect on change in rsFC over two years. We observed no apparent effect of baseline Aβ_1–42_ on change in rsFC (*p* > 0.05). For baseline p-tau_181_ however, the connection between right anterior hippocampus—right superior precuneus showed the strongest effect that survived the connection threshold of *p* < 0.001 (β = 0.44, t(62) = 3.81, [95%CI 0.208, 0.666]) but not the cluster threshold (*p* = 0.089, [95%CI_*p*_ 0.083, 0.095]). For the p-tau_181_/Aβ_1–42_ ratio, one cluster covering three ROIs of the episodic memory network was significant (*p* = 0.032, [95%CI_*p*_ 0.029, 0.035], see Fig. 4C and Supplementary Tables S5 - 6). In this cluster, a higher p-tau_181_/Aβ_1–42_ ratio was related to a steeper increase rsFC strength over two years between right anterior hippocampus—right superior precuneus (β = 0.51, t(62) = 4.19, [95%CI 0.269, 0.759]), as depicted in Fig. 4D, and between right medial parahippocampal cortex—right superior precuneus (β = 0.44, t(62) = 3.37, [95%CI 0.178, 0.696]).

There was no significant effect of any of the biomarkers of AD pathology or *APOE4* group on change in graph measures over two years (all *p* > 0.05).

### Assessment of change in cognitive performance over two years in relation to rsFC strength

There was a change in memory performance in both samples over two years. Specifically, we observed a slight increase in the RBANS delayed memory index score in sample A (A^−^T^−^ individuals) (t(90) = 3.84, *p* < 0.001, d = 0.37, [95%CI 0.174, 0.569]) from a mean score of 104 (SD = 9) to 107 (SD = 8) and in sample B (t(67) = 4.11, *p* < 0.001, d = 0.59, [95%CI 0.280, 0.890]) from a score of 102 (SD = 10) to 107 (SD = 8).

We limited our analysis on the association of rsFC (baseline or change) and memory change to the connection that showed the strongest decrease in rsFC over time in sample A (i.e. retrosplenial cortex—posterior cingulate cortex), and the connection that showed the strongest relationship with the p-tau_181_/Aβ_1–42_ ratio in sample B (i.e. anterior hippocampus—superior precuneus).

Change in rsFC over time was not significantly associated with change in memory in sample A or B (all *p* > 0.05, see Supplementary Tables S10 - 13).

**Fig. 5:**
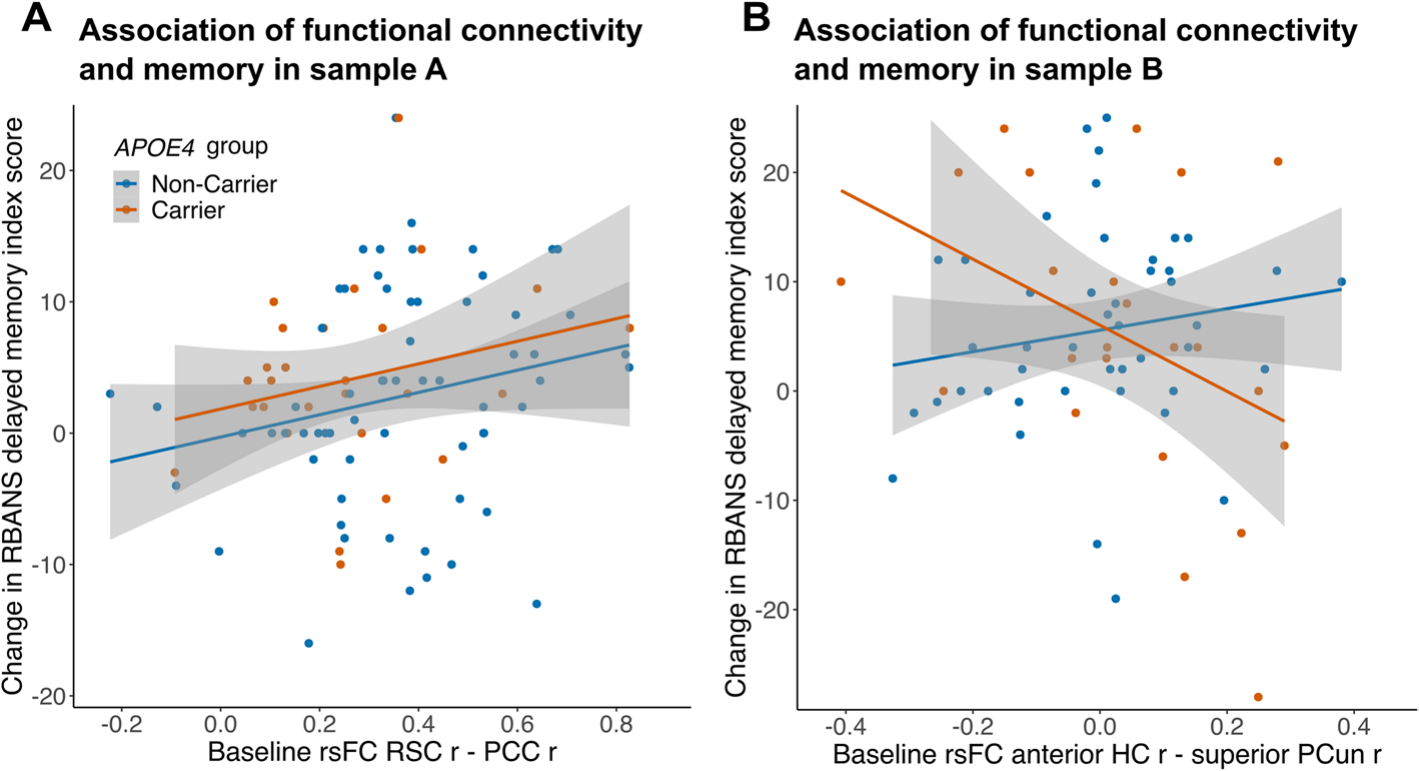
Association of baseline functional connectivity strength and change in episodic memory performance. Episodic memory performance measured with the RBANS delayed memory index score. **A** In sample A (amyloid- and tau-negative older adults), there was an association of baseline resting-state functional connectivity (rsFC) and change in episodic memory performance. There was no effect of *APOE4* group. The functional connection with the strongest decrease over time, right RSC—right PCC, was investigated. *N* = 91. **B** In sample B (older adults with available longitudinal cerebrospinal fluid (CSF) measurements), there was an interaction of baseline rsFC and *APOE4* group on change in episodic memory performance. *N* = 68. RSC = retrosplenial cortex. PCC = posterior cingulate cortex. HC = hippocampus. PCun = precuneus. RBANS = Repeatable Battery for Assessment of Neuropsychological Status. r = right

Regarding baseline rsFC in sample A, a steeper increase in memory was predicted by higher baseline rsFC between retrosplenial cortex—posterior cingulate cortex (β = 0.24, t(84) = 2.15, *p* = 0.035, [95%CI 0.018, 0.455], see Fig. 5A and Supplementary Table S7). No interaction between *APOE4* group and baseline rsFC between retrosplenial cortex—posterior cingulate cortex on change in memory was observed (*p* > 0.05). In sample B, baseline rsFC between anterior hippocampus—superior precuneus was not related to change in memory in the whole sample (*p* > 0.05). However, we found an interaction of baseline rsFC between anterior hippocampus—superior precuneus and *APOE4* group on change in memory (β = − 0.65, t(59) = − 2.54, *p* = 0.014, [95%CI − 1.169, − 0.139], see Fig. 5B and Supplementary Table S8). Running the model in both *APOE4* groups separately revealed that specifically in *APOE4* carriers, higher baseline rsFC tended to be related to less increase in memory, although the effect was only marginal (β = − 0.52, t(16) = − 2.08, *p* = 0.054, [95%CI − 1.058, 0.011], see Fig. 5B orange line and Supplementary Table S9). Due to the small sample size of 23 *APOE4* carriers, we applied additional bootstrapping with 1000 replications in this group. The generated 95%CI of the estimate [95%CI_b_ − 114.268, 4.482]) supported the finding in *APOE4* carriers (see Supplementary Figure S2). In non-carriers (*N* = 65), there was no effect of baseline rsFC on change in memory (*p* > 0.05; Fig. 5B blue line).

The p-tau_181_/Aβ_1–42_ ratio at baseline was not significantly related to change in memory performance (*p* > 0.05).

### Control analysis

First, within our control network, the visual network, there were no significant changes in rsFC over time and no effects of baseline age in sample A (all *p* > 0.05). There were no effects of AD pathology in sample B (all *p* > 0.05). Further, when including mean framewise-displacement motion estimates, all our findings remained consistent.

Second, we conducted the reported analyses regarding cognition with the RBANS attention index score instead of the delayed memory index score. We found no change in the attention score over time and no effects of baseline rsFC, change in rsFC or interactions of rsFC and *APOE4* group on change in the attention score (all *p* > 0.05, see Supplementary Tables S14 - 19).

Third, we examined whether the associations of time, baseline age, AD pathology and memory performance with rsFC strength were influenced by early structural neurodegeneration in an exploratory control analysis. Using whole bilateral hippocampus volume as a proxy of early neurodegenerative processes, we found a significant reduction thereof over two years in sample A (F(2,184) = 27.93, *p* < 0.001, η_g_^2^ = 0.0022) and sample B (F(2,134) = 12.51, *p* < 0.001, η_g_^2^ = 0.0012). Although a reduction in hippocampus volume was observed (see Supplementary Tables S20—S21 for details), the rate of decline of hippocampus volume was not associated with change in rsFC or change in episodic memory performance when included as a covariate in any of the linear models that were used in the main analyses (all *p* > 0.05). Details can be found in Supplementary Tables S22—S31.

## Discussion

### Summary

In our longitudinal study we assessed change in rsFC strength and graph measures in an episodic memory network, and in episodic memory performance in cognitively normal older adults characterized by AD biomarkers. We observed a decrease in rsFC strength over one and two years related to aging in the absence of AD pathology. This decrease was regionally specific to rsFC strength within the PMC and between the posterior hippocampus and the inferomedial precuneus. We additionally observed a decrease in global efficiency of the episodic memory network over time, which was accelerated with higher baseline age. Furthermore, we found that a steeper increase in rsFC strength over two years of the superior precuneus with the medial parahippocampal cortex and the anterior hippocampus was related to higher baseline AD pathology. Regarding episodic memory performance over time, higher baseline rsFC within the PMC was beneficial in older adults without evidence of AD pathology. However, further analyses indicated that higher baseline rsFC between the MTL and the PMC might be detrimental with respect to cognitive change for *APOE4* carriers, but not for *APOE4* non-carriers, in our sample without evidence of AD pathology. Notably, our results were specific to the episodic memory network, as there were no changes in our control network, the visual network. Moreover, our results were specific to episodic memory performance, as we observed no effect on performance in an independent attention test. Lastly, our results were independent from early neurodegeneration, as we observed no effect of hippocampal volume changes on these associations.

### Effects of aging and baseline age on episodic-network changes

Our study revealed that aging independent of AD pathology was related to a decrease in rsFC strength and global efficiency over time. Longitudinal hypoconnectivity mainly occurred within the PMC, between the posterior MTL and the inferomedial precuneus, and between the mPFC and the PMC.

Consistent with our results, previous cross-sectional studies reported lower rsFC between hippocampal and neocortical regions in older age [74–76] and emphasized age-related effects within the PMC and between the posterior MTL and the PMC in cognitively unimpaired older adults [40, 42]. A previous study that combined rsFC strength and network properties reported an association of lower rsFC and network dedifferentiation with higher age in the posteromedial network which consists of the posterior MTL and the PMC [77]. Network dedifferentiation is associated with age-related cognitive decline and refers to less distinctive neural representations of individual networks [78–80], with the DMN showing early vulnerability [81]. The observed decline in episodic-network global efficiency in our study supports these findings from whole-brain or multiple network analyses, with a loss of integration of hub nodes over time leading to more diffuse and less efficient communication within the episodic memory network.

Furthermore, we observed a steeper decrease in global efficiency of the network with higher baseline age, indicating accelerated network disconnectivity and inefficiency of the exchange of information. This was mainly due to a steeper decrease in integration of two nodes within the MTL into the episodic memory network with higher age, i.e. the perirhinal and entorhinal cortex. The third region located on the parahippcampal gyrus, the parahippocampal cortex, was further involved in a steeper decline in rsFC strength with higher baseline age. In particular, rsFC strength of the parahippocampal cortex with the perirhinal cortex and the PMC decreased more steeply over time in older individuals. The parahippocampal cortex is a major hub that functionally connects the MTL with PMC regions [82] and seems to be a key region for more pronounced decline in rsFC in older age [40, 42].

Taken together, our findings from both approaches, rsFC and graph analysis, suggest episodic-network disintegration over time as part of non-pathological “normal” aging. Further, the parahippocampal gyral regions show greater age-related vulnerability to network disintegration [83], shifting the parahippocampal gyrus into focus for identifying the earliest age-related and AD-pathology independent functional changes.

### Effect of Alzheimer’s pathology on on episodic-network changes

While we observed little change in AD pathology measured via CSF over two years, we report a steeper increase in rsFC of the anterior hippocampus with the superior precuneus and of the parahippocampal cortex with the superior precuneus over two years with a higher baseline p-tau_181_/Aβ_1–42_ ratio.

Consistently, a previous cross-sectional tau-PET study reported an association of higher rsFC strength between the hippocampus and the retrosplenial cortex and higher PMC tau burden in cognitively unimpaired older adults, however, this relationship was independent from Aβ burden [30]. In contrast, another cross-sectional study reported lower rsFC strength between the anterior hippocampus and the PMC with higher CSF p-tau_181_ burden in amyloid-positive cognitively unimpaired older adults [23]. While our results also suggest a combined effect of CSF-measured Aβ and tau, Berron and colleagues report hypoconnectivity instead of hyperconnectivity with more pathology. There are several differences that could contribute to these diverging results, most importantly, their sample was approximately 10 years older than ours. Interestingly, while their sample consisted exclusively of amyloid-positive individuals, the mean CSF p-tau_181_ burden at baseline was lower in their sample than in our sample B. Caution should, however, be taken when comparing these factors due to potentially differing CSF processing and the cross-sectional nature of their study.

While tau-PET can quantify and localize tau that is aggregated in neurofibrillary tangles, CSF tau measures provide information about earlier processes. Soluble CSF p-tau_181_ serves as a marker of abnormal tau phosphorylation, a process that precedes the formation of tangles and is closely associated with Aβ plaques [84, 85]. Recent findings suggest that CSF p-tau may drive the accumulation of tau tangles related to CSF Aβ pathology [86]. Hyperphosphorylated tau is produced and secreted by neurons that, while still functional, are already compromised by Aβ accumulation and neuronal hyperexcitation [87–89]. CSF Aβ_1–42_ is a marker for soluble Aβ peptides that are prone to aggregation into plaques, with lower values indicating one of the earliest processes of AD [90]. Our finding for the p-tau_181_/Aβ_1–42_ ratio, but not p-tau_181_ (did not reach the cluster threshold) or Aβ_1–42_ individually, suggests that a combination of both pathology CSF-markers showing abnormalities impacts change in rsFC. Specifically, elevated hyperphosphorylated tau could start to impact the trajectory of rsFC strength between the MTL and the PMC when abnormal Aβ levels are present, while tau tangles could impact rsFC independently of Aβ further down the line [30].

There is accumulating evidence that early amyloid in the PMC might lead to local hyperactivation and aberrant FC of the MTL due to the lack of neocortical inhibition [11]. A recent study that used dynamic causal modeling (DCM) during an fMRI memory task showed reduced inhibition from PMC to MTL regions with increasing amyloid burden, this directed “hyperexcitation” in turn predicted MTL tau accumulation [91]. Amyloid-induced tau spread from the MTL to functionally connected regions [22, 24, 86, 92] could then contribute to a vicious cycle of pathology accumulation, aberrant activity and FC [2, 27, 32]. Consistently, higher and increasing precuneus activity during memory retrieval was linked to subsequent higher global Aβ-PET burden in a recent study within the PREVENT-AD cohort [73].

Overall, our results fit into this framework and suggest that the observed hyperconnectivity between the MTL and the PMC may be driven by early pathological processes, specifically elevated p-tau_181_ levels in combination with reduced Aβ_1–42_ levels in CSF.

### Effects of functional connectivity strength and *APOE* genotype on change in cognition

We observed a change in episodic memory performance over two years in both samples with a slight increase in performance, possibly reflecting practise effects [93]. Practice effects are commonly observed in cognitively unimpaired older adults and diminished practice effects are associated with higher AD pathology burden and cognitive decline, suggesting that the variation in improvement in performance is a meaningful measure [94, 95]. Higher baseline rsFC strength within the PMC seemed to be beneficial for longitudinal cognition regardless of *APOE4* genotype in older adults without AD pathology. This is consistent with previous cross-sectional and longitudinal findings that have shown that memory-task activity and rsFC in older adults, which more closely resemble activity and rsFC of younger adults, are beneficial [23, 96–98]. In younger adults, however, no associations were found between cognitive performance and FC during rest and task in a previous study, suggesting that FC strength may play a more significant role in older age [99].

We further observed differential relationships on cognition for baseline rsFC between the MTL and the PMC depending on *APOE* genotype. Specifically, higher baseline rsFC strength between the anterior hippocampus and the superior precuneus tended to be detrimental in *APOE4* carriers, but not in *APOE4* non-carriers. We also observed more AD pathology in *APOE4* carriers, which was, however, itself not related to memory performance. Furthermore, there were no significant differences in rsFC or cognition between *APOE4* groups, as reported before [100, 101]. The differing FC—behavior relationship we observed could be due to subtle neurobiological changes related to early AD-pathology in *APOE4* carriers. Previous cross-sectional findings reported mixed results regarding the relationship between MTL—PMC rsFC and memory performance in dependence of *APOE4* genotype*.* In some studies, better memory performance was associated with higher rsFC between the hippocampus and the PMC in older age regardless of *APOE* genotype [102, 103]. On the contrary, better episodic memory performance was related to lower rsFC of the temporal DMN only in *APOE4* carriers [104]. We did not observe an effect of change in rsFC on change in memory performance over time, and no interaction of change in rsFC with *APOE*, age or AD pathology.

In summary, our findings do not support the hypothesis that hyperconnectivity between the anterior hippocampus and the superior precuneus is beneficial or compensatory in the presence of AD risk as it was not related positively but rather negatively to future change in memory performance in *APOE4* carriers [105]. However, the effect of change in FC on trajectories of cognition should be further investigated in long-term studies.

### Limitations

First, we acknowledge that when investigating resting-state fMRI, there is limited information on cognitive processes during scanning. However, rsFC can provide valuable insights into brain dynamics relevant for cognition [101] and functional changes have been reported in cognitively unimpaired older adults both during cognitive tasks [106] and at rest [101]. Future studies could directly extend our findings by assessing FC during memory-task performance and relate these findings to longitudinal amyloid, tau, behavior, and the *APOE* genotype.

Second, our sample size was limited and predefined, as we used an existing dataset. Given the limitations in reliability of rs studies [107], a larger sample size may be necessary to achieve adequate power. However, we preregistered our a-priori hypotheses and methods to strengthen our study. Nonetheless, our results should be interpreted with caution and warrant further replication. Further research could additionally focus on more fine-grained regional differences and investigate hippocampal and PMC subregions.

Third, we note that the relatively small network size (16 nodes) may limit the generalizability of our graph-analyses results to other cohorts, and caution is advised when comparing these findings with studies that investigated larger or more complex brain networks.

Fourth, the PREVENT-AD cohort consists of primarily white, highly educated female participants, thus representing only a sample of the general population. Future research should aim to replicate our findings in more ethnically and sociodemographically diverse cohorts.

Fifth, our sample of A^−^T^−^ participants shows variation in their sub-threshold levels of amyloid and tau, ranging from no detectable pathology to slightly below cut-off. Further, A^−^T^−^ status was determined via two different measures; PET and CSF. Despite these limitations, our study contributes much needed longitudinal findings, as AD biomarkers, especially for tau pathology, were often not available in previous aging studies.

## Conclusion

In summary, our results highlight differential changes in region-specific longitudinal rsFC strength of the episodic memory network for amyloid- and tau-negative “normal” aging and early AD pathology. A decrease in rsFC within the PMC occurs over time, however, higher rsFC within the PMC appears to be beneficial for episodic memory performance in non-pathological “normal” aging. The parahippocampal gyrus seems to be especially affected by decrease in rsFC and global efficiency in older age. Higher AD pathology is related to a steeper increase in rsFC strength between the MTL and the PMC, specifically between the anterior hippocampus and the superior precuneus. These findings shed light on early hyperconnectivity between the MTL and the PMC with AD pathology, which might already be disadvantageous for episodic memory performance in *APOE4* carriers. Thus, our study emphasizes that higher or increasing rsFC strength should not be termed beneficial or detrimental per se, but the impacted brain areas, AD biomarkers and risk factors need to be considered carefully.

## Supplementary Information

Supplementary Material 1

## Data Availability

Some of the data used in this manuscript is openly available at https://openpreventad.loris.ca. The remaining data can be shared upon approval by the scientific committee at the Centre for Studies on Prevention of Alzheimer’s Disease (StoP-AD) at the Douglas Mental Health University Institute after registration at https://registeredpreventad.loris.ca. Code for PET preprocessing is available at https://github.com/villeneuvelab/vlpp and code for statistical analysis is available at https://github.com/fislarissa/MTL_PMC_longitudinal_rsFC.

## Abbreviations

AD: Alzheimer’s Disease
CI: Confidence Interval
CSF: Cerebrospinal Fluid
DZNE: German Centre for Neurodegenerative Diseases
EC: Entorhinal Cortex
fMRI: Functional Magnetic Resonance Imaging
mPFC: Medial Prefrontal Cortex
MTL: Medial Temporal Lobe
PCC: Posterior Cingulate Cortex
PET: Positron Emission Tomography
PHC: Parahippocampal Cortex
PMC: Posteromedial Cortex
PRC: Perirhinal Cortex
RBANS: Repeatable Battery for Assessment of Neuropsychological Status
RSC: Retrosplenial Cortex
rsFC: Resting-State Functional Connectivity
SUVR: Standardised uptake value ratios
